# Case of Incised Wound of the Abdomen, with Transverse Division of the Small Intestine in Two Places, and Division of the Mesenteric Artery

**Published:** 1868-12

**Authors:** James L. Ord

**Affiliations:** Santa Barbara


### Case of Incised Wound of the Abdomen, with Transverse Division of the Small Intestine in two places, and division of the Mesenteric Artery.

BY JAMES L. ORD, M. D., SANTA BARBARA.

October 7th, 1867.—Was called to see B. O., a native Californian, aged thirty, who had received an incised wound in the left iliac region, over the spinous process of ilium. Arrived about two hours after he was wounded. Found the small intestines protruding enough to fill a hat, and cut in two places transversely, and a large branch of the superior mesenteric artery divided and bleeding profusely. The bowels were red and much congested; some of the forces had exuded from the intestines. Tied the artery with white silk, and sewed up the intestines with common *sewing cotton*, and a fine needle; gradually reduced the bowel.

In tying the artery and sewing the gut, left about four inches of the thread, intending to leave the ends out, but in reducing the bowel they went in together. The external wound was partially closed by two sutures, leaving the lower part open, so as to let out the blood, etc., that might have collected in the cavity of the abdomen. There was considerable time occupied in reducing the bowels; as the opening was small, a little of either end was reduced at a time. No chloroform was used.

Gave Dover's powder, gr. xx, there being considerable pain and tenderness of the abdomen.

Next day gave hydrarg. sub. mur., gr. xx; there still being much abdominal pain on breathing.

October 9th.—Saw the man to-day; doing well; pulse 86; breathing, 36; not as much abdominal pain on breathing; gave hydrarg. and tart. antim. to check peritonitis, and act on the bowels; considerable sanious discharge from the wound; gave no food, except water and corn meal gruel on the second day.

October 11th.—Had an operation from his bowels yesterday; little or no abdominal inflammation; appetite improving; ordered his diet to be increased; discharge from the wound still great; yesterday gave sulph. magnes. zss, in divided doses.

October 15th.—Doing well; asked to get up; external wound smaller; discharge not so great; little or no tenderness on pressure of the abdomen, and no pain in breathing; at night complains of some pain which disturbs his sleep; gave sulph. morph. gr. j, at night; requested the attendants to notice if any pieces of thread pass the bowels.

November 10th.—This man rode to town to-day on horseback, distance about five miles, to report himself perfectly recovered. His attendants did not see anything of the pieces of the threads that were used in sewing up the wounds, and so I think they must have been absorbed.

October, 1868.—This man has since died (September, 1868,) with phthisis; was not able to make a post mortem, being absent at the time of his death.—*California Medical Gazette.*

